# Changes in Striatal Dopamine Release Associated with Human Motor-Skill Acquisition

**DOI:** 10.1371/journal.pone.0031728

**Published:** 2012-02-15

**Authors:** Shoji Kawashima, Yoshino Ueki, Takashi Kato, Noriyuki Matsukawa, Tatsuya Mima, Mark Hallett, Kengo Ito, Kosei Ojika

**Affiliations:** 1 Department of Neurology and Neuroscience, Nagoya City University Graduate School of Medical Science, Mizuho-ku, Nagoya, Japan; 2 Department of Brain Science and Molecular Imaging, Research Institute, National Center for Geriatrics and Gerontology, Morioka, Obu, Aichi Prefecture, Japan; 3 Human Brain Research Center, Kyoto University Graduate School of Medicine, Sakyo-ku, Kyoto, Japan; 4 Human Motor Control Section, National Institutes of Health, Bethesda, Maryland, United States of America; Universidad de Castilla-La Mancha, Spain

## Abstract

The acquisition of new motor skills is essential throughout daily life and involves the processes of learning new motor sequence and encoding elementary aspects of new movement. Although previous animal studies have suggested a functional importance for striatal dopamine release in the learning of new motor sequence, its role in encoding elementary aspects of new movement has not yet been investigated. To elucidate this, we investigated changes in striatal dopamine levels during initial skill-training (Day 1) compared with acquired conditions (Day 2) using ^11^C-raclopride positron-emission tomography. Ten volunteers learned to perform brisk contractions using their non-dominant left thumbs with the aid of visual feedback. On Day 1, the mean acceleration of each session was improved through repeated training sessions until performance neared asymptotic levels, while improved motor performance was retained from the beginning on Day 2. The ^11^C-raclopride binding potential (BP) in the right putamen was reduced during initial skill-training compared with under acquired conditions. Moreover, voxel-wise analysis revealed that ^11^C-raclopride BP was particularly reduced in the right antero-dorsal to the lateral part of the putamen. Based on findings from previous fMRI studies that show a gradual shift of activation within the striatum during the initial processing of motor learning, striatal dopamine may play a role in the dynamic cortico-striatal activation during encoding of new motor memory in skill acquisition.

## Introduction

Motor skill learning is defined as a change in motor performance with practice and includes a number of aspects such as increasing the repertoire of motor behaviour and maintenance of acquired behaviour over a period of time [1] . If a point-to point movement is made faster and with greater accuracy through practice, there results in a learning process, recognized as a new skill acquisition [2], [3], [4], [5]. Such motor skill acquisition is essential in daily life. It is based on the formation of order of complex movements with sequential elements (learning new motor sequence) and reconstruction of muscle control of isolated movement (encoding elementary aspects of movement) [6], [7]. Many functional imaging studies revealed that the neural basis of the motor skill learning is attributed to different portions of the brain including the motor cortices, cerebellum and basal ganglia [8], [9], [10], [11].

Dopaminergic signals in the striatum and motor cortex play essential roles in the induction of synaptic plasticity and motor skill acquisition. Administration of a D1 receptor antagonist to the striatum previously resulted in impaired motor skill acquisition [12] [13], while ^11^C-raclopride positron emission tomography (PET) showed dopamine release in the striatum during new motor sequence learning [14]. The motor cortex is also associated with encoding elementary aspects of movement such as dynamic acceleration and force [15], [16], [17].

Muellbacher and colleagues previously carried out a transcranial magnetic stimulation (TMS) study in which subjects rapidly learned how to optimize ballistic thumb flexion with the aid of visual feedback, as indicated by increased thumb acceleration. The simple repetitive movements changed into an acquired motor skill after 60 minutes of training. The acquisition of new motor skills was shown to be associated with the early consolidation of motor memory, the memory stabilization from interference by repetitive TMS, causing rapid induction of motor cortical plasticity. Evidence indicated that encoding elementary aspects of movement can be related to the formation of new motor memory [18], [19]. However, it remains unclear whether striatal dopamine is associated with encoding of new motor memory during skill acquisition.

The aim of the present study, therefore, was to investigate whether striatal dopamine is related to the intrinsic processing of new motor memory, dependent on the time course of training. We examined striatal intrinsic dopamine levels as measured by ^11^C-raclopride PET during the skill acquisition task developed by Muellbacher on Day 1 (initial skill-training) and Day 2 (acquired conditions). Our hypothesis was that striatal dopamine levels would change in association with encoding of new motor memory during skill acquisition.

## Materials and Methods

### Subjects

Ten healthy volunteers (six males, four females; mean age ± standard deviation [SD] = 68.8±2.7 years) with no history of neurological or psychiatric disorders were enrolled in the study. All subjects were right-handed according to the Edinburgh Inventory (Oldfield, 1970). All participants provided written and informed consent in accordance with the dictates of the trust ethics committee of Nagoya-City University Hospitals, Nagoya, Japan and the National Centre for Geriatrics and Gerontology, Obu City, Japan. The ethics committee of Nagoya-City University Hospitals and the National Centre for Geriatrics and Gerontology specifically approved this protocol (protocol number 46-08-0002).

### Experimental procedure

To elucidate potential changes in striatal dopamine release associated with differences in the processing of motor memory dependent on the time course of skill-training, ^11^C-raclopride positron-emission tomography (PET) scanning was performed on two separate days, two weeks apart (Day 1; initial skill-training, Day 2; acquired). This technique detects subtle differences in the amounts of dopamine released under different conditions [20], [21], [22]. Based on previous^11^C-raclopride PET studies, the task was started 5 min prior to the injection of ^11^C-raclopride to maintain detection sensitivity throughout the duration of the scan [22], [23].

**Figure 1:**
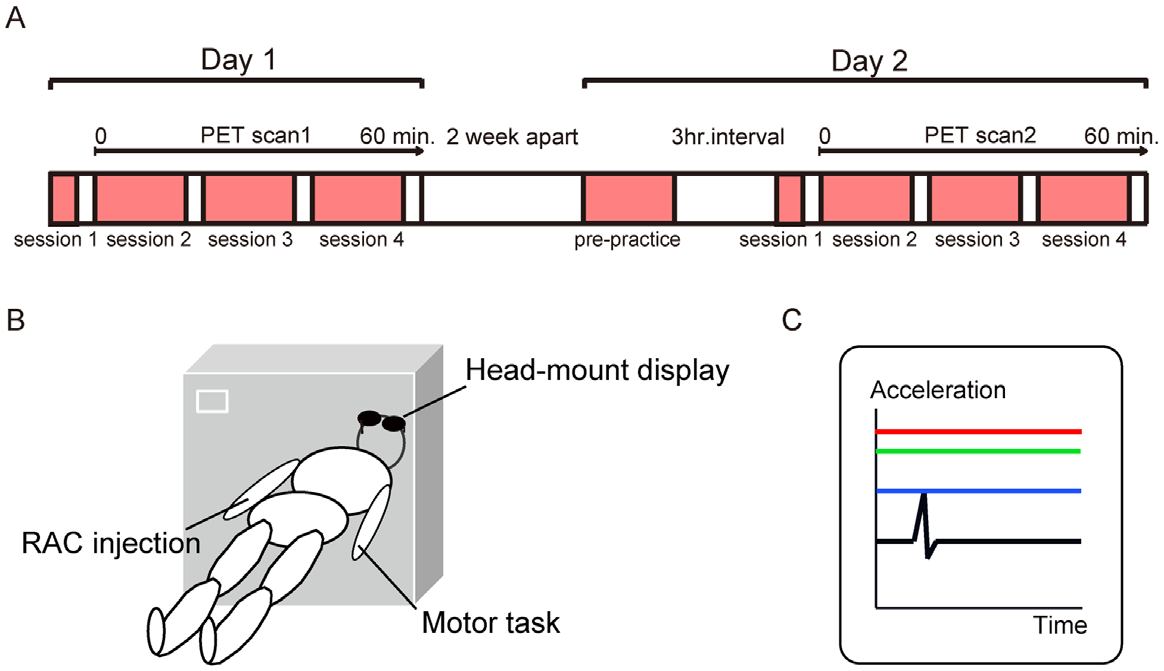
Experimental procedures. (A) Time schedule of experiments. Subjects were scanned for evaluating striatal dopamine levels using ^11^C-raclopride PET on Day 1 (initial skill-training condition) and Day 2 (acquired condition). (B) ^11^C-raclopride PET scanning. ^11^C-raclopride (555 MBq) was injected into the right vein just before the emission data was acquired. They practiced rapid contraction of their left thumb via visual feedback during PET scanning. (C) Display seen by subjects during motor practice. Three coloured horizontal lines were graphically presented to help subjects understand the quality of their performance: peak acceleration of the present movement (blue), mean peak acceleration of all previous trials within a block (green) and maximum peak acceleration of all previous trials within a block (red).

During Day 1, subjects rapidly learned how to effectively contract their left flexor pollicis brevis muscles with the aid of visual feedback. On Day 1, they performed one block of motor practice outside the scanner (session 1) then completed a further six blocks while they underwent PET scanning (session 2, blocks 2–3; session 3, blocks 4–5; session 4, blocks 6–7). Each block of motor practice included 60 movements in block 1 and 120 movements in blocks 2–7. A 3-min rest period was provided between each block, and it took 3 min in block 1 and 6 min in each block 2–7 to complete. There was no interim practice between Day 1 and Day 2. Two weeks after Day 1, on Day 2, the same subjects performed four blocks of motor prepractice (block 1 and blocks 2–4) 3 hours before the PET scan in order to retrieve the skill. In the acquired condition, participants then performed seven blocks of the motor task (sessions 1–4), as described for initial skill-training conditions: that is, they performed session 1 outside the scanner and then completed sessions 2–4 while they underwent PET scanning (Figure 1A, 1B).

The difference in levels of striatal dopamine released between the two conditions represents the encoding of new motor memory during skill acquisition, if the motor skill was retained in the acquired condition.

### Behavioural task

The task was modified from the established motor skill-acquisition protocol [18], [19], [24] and consisted of repeated rapid contraction of the left thumb. Subjects rapidly learned how to optimise rapid finger movements with the aid of visual feedback.

Subjects practised rapid contraction of their left flexor pollicis brevis muscles to the beat of a metronome every 3 s. The non-dominant hand was chosen to ensure that the task involved motor learning [25]. Each block of motor practice included 60 movements in block 1 and 120 movements in blocks 2–7. The change in sequential one-dimensional acceleration of the left thumb during each contraction was recorded with a piezoelectric accelerometer mounted on the proximal phalanx (model 25A, Isotron PE Accelerometer, 4.575 mVg^−1^ sensitivity, Endevco, San Juan Capistrano, CA). The signal was amplified with a battery-powered low-noise signal conditioner (model 4416B Isotron Signal Conditioner, Endevco) and digitized with a PCI-MIO-16E4 board (National Instruments, Austin, TX) at a rate of 2,000 Hz. Peak acceleration was recorded precisely using 6,000 sampling points within 3 s.

Subjects were asked to perform the metronome-paced muscle contraction as fast as possible with the aid of visual feedback. Real-time acceleration was graphically displayed on the monitor for 1 s immediately after the end of movement. Data were graphically presented to subjects as three coloured horizontal lines to help them understand the quality of their performance: peak acceleration of the present movement (blue), mean peak acceleration of all previous trials within a block (green) and maximum peak acceleration of all previous trials within a block (red). Subjects were instructed to focus on elevating the blue line above the green line (Figure 1C). The increase of peak acceleration was displayed as the elevation of the green line. Data from each behavioural trial were stored on a laboratory computer for offline analysis.

The maximal peak force between the index finger and the thumb was also measured five times with a pinch gauge (in kgf) (JAMAR Hydraulic Pinch Gauge, PC-7498-05; Sakai Co, Japan) immediately before session 1 and after session 3 for both initial skill-training and acquired conditions.

### Behavioural data analysis

To assess the quality of motor-skill performance, the mean peak acceleration of the 60 or 120 executed movements in each block was calculated (in cm/s^2^). The first 10 trials in session 1 were excluded from analysis because of incomplete data acquisition and high variability. Mean values for the remainder of sessions 1–4 were therefore analysed.

We hypothesized that the motor performance in the Day 1 would improve with repeat practice to ultimately reach plateau level, whereas, any more performance improvement would not occur in the Day 2. To confirm behavioural changes reflecting skill acquisition in the present protocol, mean accelerations were compared using one-way repeated-measures analyses of variance (ANOVA), with the time of sessions 1–4 as a within-subject variable, followed by a *post-hoc* paired *t*-test. For the acquired condition, we evaluated whether the mean acceleration was retained from start to finish using one-way repeated-measures ANOVA. Missing data due to accelerometer failure during the PET scan were linear-interpolated.

### Imaging data acquisition and analysis

Volumetric anatomical magnetic resonance imaging (MRI) of the brain was carried out with a 1.5-Tesla scanner, using a T1-weighted inversion-recovery fast-spoiled gradient-echo sequence (Phillips Intera 1.5T; slice thickness, 1 mm; transverse plane; repetition time, 8.3 ms; echo time, 3.8; flip angle = 30°; matrix size, 256×256).

^11^C-raclopride PET scans were performed using an ECAT EXACT HR tomograph (CTI/Siemens, South Iselin, NJ) in three-dimensional acquisition mode, which yielded 47 simultaneous planes, with an inter-slice spacing of 3.125 mm, an axial full-width at half-maximum resolution of 4.8 mm and an in-plane resolution of 4.0–3.9 mm at the centre. The subject was positioned in the scanner so that the entire brain was within the field of view. The head position was maintained using moulded foam headrests. To correct for tissue attenuation of 511 keV annihilation radiation, two-dimensional transmission scanning was performed for 10 min prior to the tracer injection using a retractable 68Ga/68Ge source. ^11^C-raclopride (555 MBq) was injected into the right antecubital vein over a period of 30 s, and emission data were acquired over a period of 60 min in 19 sequential frames of progressively increasing duration. The injection was performed between 15:00 and 16:00.

Two analytical methods were used to estimate changes in striatum dopamine release: a demonstration of changes in ^11^C-raclopride binding using the region of interest (ROI) approach, and a voxel-based analysis using statistical parametric mapping [26].

### ROI analysis

^11^C-raclopride PET data were pre-processed and analysed by a neuroradiologist using PMOD software (PMOD Technologies, Zurich, Switzerland). The dynamic PET frames for the two conditions and the MRI T1 structural images were initially imported into PMOD, and image volumes were reoriented in accordance with the inter-hemispheric fissure and inter-commissural plane. The ROIs were then traced around the caudate and putamen of the bilateral hemispheres on each subject's MRI T1 image for all planes in which these structures were clearly defined. Reference regions were traced on the bilateral cerebellum as two elliptic regions placed over the cerebellar hemisphere.

MRI T1 images and PET scans of the two conditions were rigidly matched by the shift and rotation of spatial coordinates. In this co-registration process, we performed intra-modality matching for PET scans, and the MRI T1 images were then spatially transformed to PET scans using the automatic cross-modality matching method (with trilinear interpolation, an 8-mm sample rate and the Powell minimization method).

The binding potential (BP) was calculated from the radioactivity concentration ratios in regions with and without specific receptor binding. The cerebellum was used as a receptor-less reference region because of its paucity of dopamine receptors. In the present study, ^11^C-raclopride BP was computed using regional time-activity curves, which indicated changes in radioactivity concentration. By applying the ROIs and reference regions as volumes of interest (VOIs), time-activity curves were obtained for receptor-rich (putamen and caudate) and receptor-less (cerebellum) regions [27]. BP was computed using Logan reference-region graphical analysis [28]. A reduction in ^11^C-raclopride BP indicated an increase in extra-cellular dopamine concentration [29].

Changes in ^11^C-raclopride BP were evaluated using a repeated-measure ANOVA, with condition (Day 1 and Day 2) as a within-subject variable and place (right putamen, left putamen, right caudate and left caudate) as a between-group variable. The Greenhouse-Geisser method was used to correct for nonsphericity. If the effect was significant, a *post-hoc* paired *t*-test was performed on the data.

### Voxel-by-voxel analysis

Striatal and cerebellar VOIs were created using MRIcro software (Version 1.40.1; http://www.sph.sc.edu/comd/rorden; Chris Rorden, University of South Carolina, Columbia, SC) on MRI T1 images. VOIs and MRI T1 images for each subject were co-registered to the integrated PET images, which were produced by summing the frames from the first 5 min of the PET scanning in the skill-acquisition condition. Co-registered VOIs were then used to obtain regional time-activity curves for receptor-rich and receptor-less regions within PMOD. These curves were incorporated into the pixel-by-pixel calculation of BP, then parametric BP images were created.

Co-registered MRI T1 images were transformed into standardized Montreal Neurological Institute (MNI) stereotactic space (http://www.bic.mni.mcgill.ca) using Statistical Parametric Mapping 5 (SPM5; Wellcome Department of Imaging Neuroscience; freely available at http://www.fil.ion.ucl.ac.uk/spm) implemented within Matlab R2006. In the process of spatial normalization using SPM5 default parameters, the transformation matrix obtained from this step was applied to the parametric BP images, placing all BP images in MNI space. MRI T1 images and striatal VOIs were directly transformed into MNI space, then the averaged image of all subjects' normalized VOIs was created. BP images and the averaged striatal VOI were resliced to a final voxel size of 3×3×3 mm, and smoothed with an 8-mm full-width at half maximum Gaussian kernel.

In order to elucidate sub-regions of the striatum which indicated significant change between the two conditions, voxel-wise paired *t*-tests between the initial skill training and acquired conditions were conducted in SPM5 as follows: initial skill training – BP<acquired – BP, and acquired – BP<initial skill training– BP, without a global normalization procedure. To limit analysis within the striatum, the striatal VOI was used for mask image in the process of voxel-wise comparisons. The threshold of significance was set at *P*<0.05.

## Results

### Behaviour

The mean acceleration showed significant repetition-related improvements and a constant value of learning was attained during initial skill-training, but not the acquired condition, with the improved performance being retained and over-learned at the start of the latter condition.

**Figure 2:**
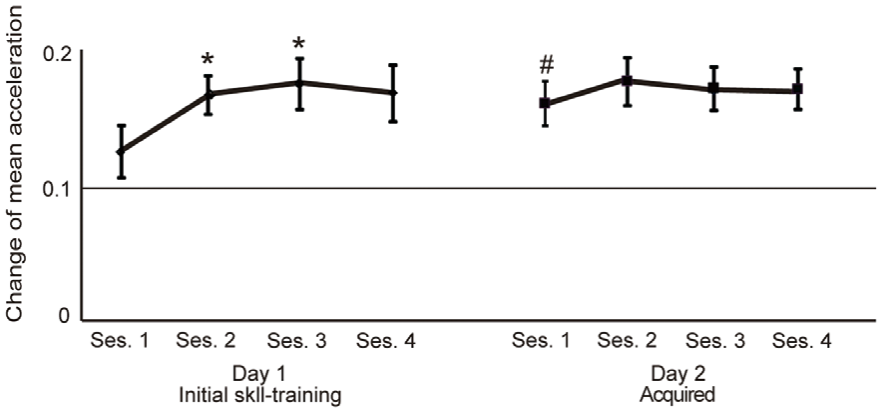
Mean acceleration changes in initial skill-training and acquired conditions. In initial skill-training, the mean accelerations were significantly increased in sessions 2 and 3 compared with session 1, whereas the mean acceleration in session 4 did not reach statistical significance. For the baseline, *t*-testing revealed a significant increase in mean acceleration for session 1 in the acquired condition compared with initial skill-training. In the acquired condition, no additional gains in acceleration were observed in sessions 2–4. Y axis indicates the ratio of mean accelerations compared with baseline of session1 on Day 1. Error bars show the SEM. *P<0.05, #*P*<0.01.

Changes in the mean acceleration of sessions 1–4 were separately compared using one-way repeated-measures ANOVA, which demonstrated a significant effect of time (*P = 0.03). The mean (± standard error of the mean [SEM]) accelerations in session 2 (1,650±120 cm/s^2^) and session 3 (1,790±170 cm/s^2^) were significantly increased compared with session 1 (1,260±130 cm/s^2^) (*P = 0.02 in session 2 and *P = 0.05 in session 3), whereas the mean acceleration in session 4 did not reach significance (1,650±170 cm/s^2^) (*P* = 0.15) (Figure 2). This finding was in agreement with previously reported data on the learning-related improvement in elementary movement, which showed significant gains in acceleration until the end of a 60-min learning period [19].

**Figure 3:**
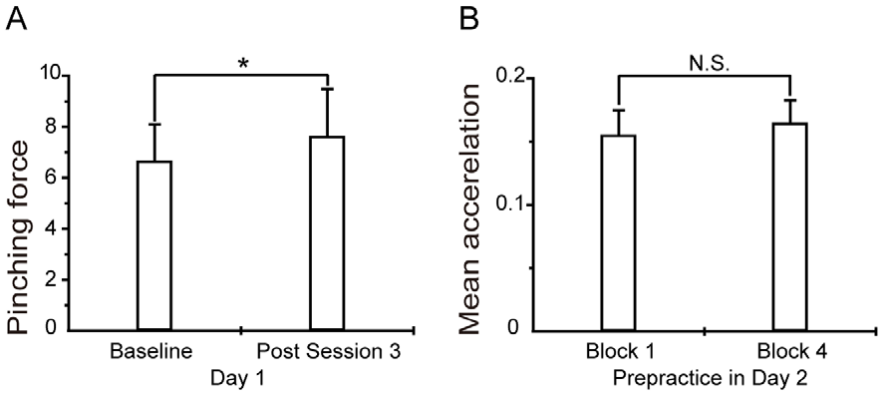
Mean pinching force changes in initial skill-training on Day 1 and mean acceleration changes in prepractice on Day 2. (A) Changes of the mean pinching force in the initial skill training between baseline (before session 1) and post session 3. Y axis indicates mean pinching force (kg). Error bars show the SD. *P<0.05. (B) Mean accelerations in the motor prepractice between block 1 and block 4. Y axis indicates mean accelerations (cm/s^2^). Error bars show the SEM. N.S. not significant.

By contrast, in the acquired condition, one-way repeated measures ANOVA demonstrated no effect of time (*P* = 0.43), without additional gains in acceleration (1,620±160 cm/s^2^ in session 1, 1,810±170 cm/s^2^ in session 2, 1,750±150 cm/s^2^ in session 3 and 1,740±140 cm/s^2^ in session 4). Moreover, the mean pinching force of the post session 3 was significantly increased compared with the baseline (before session 1) in the initial skill-training (mean ± SD 6.6±1.5 kg for baseline and 7.6±1.9 kg for post session 3, **P = 0.02, examined by paired *t*-test) (Figure 3A). By contrast, it was not changed in the acquired condition (7.2±2.8 kg before, 7.3±2.1 kg after, *P* = 0.8).

For the baseline of session 1, the mean acceleration was significantly increased in the acquired condition of Day 2 (27.2%±3.4) compared with initial skill-training, suggesting that the improved performance was retained in the former (**P = 0.007, examined by *t*-test). In the motor prepractice in Day 2, one-way repeated measures ANOVA demonstrated no effect of time (*P* = 0.93), without additional gains in acceleration (1,550±200 cm/s^2^ in session 1, 1,660±160 cm/s^2^ in session 2, 1,700±190 cm/s^2^ in session 3 and 1,640±180 cm/s^2^ in session 4). The mean acceleration did not differ significantly between block 1 and block 4 (*P* = 0.56) (Figure 3B). In addition, the mean acceleration did not differ significantly between session 4 of Day 1 and session 1 of the acquired condition of Day 2 (*P* = 0.76), and between session 4 of Day 1 and block 1 of the motor prepractice in Day 2 (*P* = 0.49). This finding supports the notion that the improved motor performance obtained during training in Day 1 was retained in Day 2, and that the performance was no further improved after the prepractice of Day 2.

Comparison of the mean acceleration in sessions 1–4 between initial skill-training and the acquired condition showed no statistical difference (mean ± SEM = 1,590±150 cm/s^2^ versus 1,730±160 cm/s^2^, *P* = 0.2, examined by *t*-test), suggesting that the motor performance itself did not differ between the two conditions.

### ^11^C-raclopride PET study

**Figure 4:**
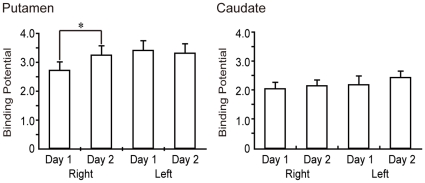
^11^C-raclopride BP changes in the bilateral putamen and caudate nucleus between the two conditions. In the right putamen, ^11^C-raclopride BP was significantly reduced during initial skill-training compared with the acquired condition, whereas no significant reduction was observed in the left putamen. In the bilateral caudate, no significant changes were observed between the two conditions. The mean (± SEM) BP during initial skill-training was 2.72±0.86 in the right putamen and 3.42±0.99 in the left putamen.

In the ROI analysis of ^11^C-raclopride BP, repeated measures ANOVA demonstrated a significant main effect of place (*P = 0.005) and condition (*P = 0.02), as well as an interaction between condition and place (*P = 0.04). The *post-hoc* paired t-test revealed that^11^C-raclopride BP was significantly reduced on Day 1 compared with Day 2 in the right putamen (*P = 0.02, F(1,9) = 8.0), but not in the left putamen (*P* = 0.42, F(1,9) = 0.07), right caudate (*P* = 0.98, F(1,9) = 0.001) or left caudate (*P* = 0.47, F(1,9) = 0.59) (Figure 4). Thus, changes in striatal dopamine release were only observed within the right putamen.

**Figure 5:**
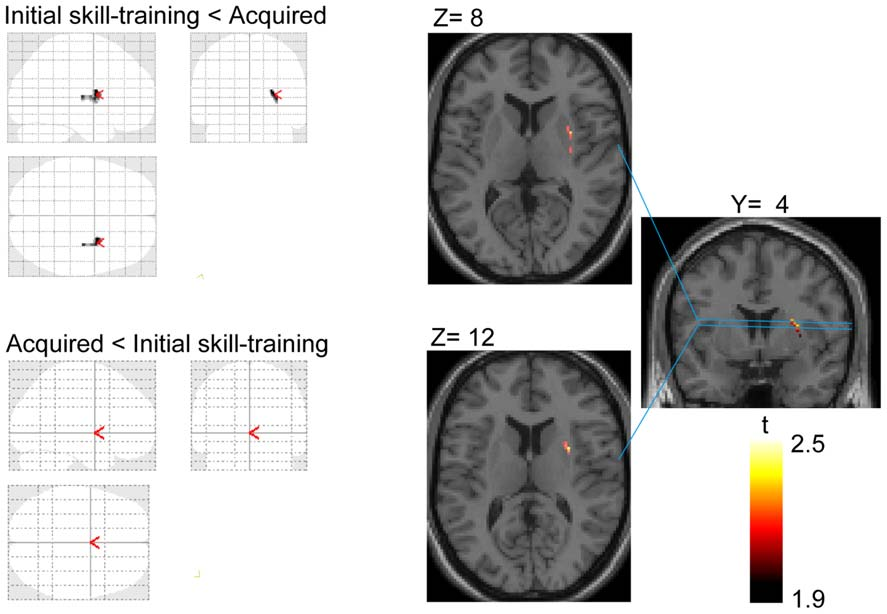
^11^C-raclopride BP changes in sub-regions of the striatum between the two conditions. Left panel shows the grass brain map of the voxel-based analysis of ^11^C-raclopride BP change (upper figure: initial skill-training condition < acquired condition and lower figure: acquired condition < initial skill-training condition). Right panel shows coronal and axial sections of the statistical parametric map of ^11^C-raclopride BP change in the initial skill-training condition versus acquired condition overlaying the MRI T1 image in stereotaxic space. Right side image corresponds to right side brain. The displayed cluster shows the significant area of decreased ^11^C-raclopride BP in the right antero-dorsal and lateral part of the putamen. The peak coordinate in the right putamen was located at X = 30, Y = 4, Z = 12. No BP change was observed in the left putamen.

Voxel-wise analysis was performed to elucidate sub-regions of the striatum that demonstrated a significant change between the two conditions. ^11^C-raclopride BP in the right antero-dorsal to lateral part of the putamen was reduced during initial skill training compared with the acquired condition (initial skill-training – BP<well-learned – BP) (Figure 5). The peak coordinate of the area of BP reduction was within the anterior part of the putamen (X = 30, Y = 4, Z = 12). No such reduction in BP was observed in the acquired compared with the initial skill training conditions (acquired – BP<initial skill-training– BP), and no significant BP changes were detected in the bilateral caudate and left putamen.

## Discussion

This study revealed a reduction in ^11^C-raclopride BP within the right putamen during initial skill-training compared with acquired conditions, which was associated with increased acceleration of left-thumb movement. Voxel-wise analysis confirmed this finding, and further localised the area of change to the right antero-dorsal to lateral part of the putamen.

### Role of striatal dopamine release during motor skill acquisition

During initial skill-training, the mean acceleration of each session was improved through repeated training sessions (session 2–3) until performance approached asymptotic levels (session 4), whereas the improved motor performance was retained from the beginning of the acquired condition. The pinching force results revealed a similar tendency in accordance with previous reports [18], [19]. In the present study, although subjects performed a 3-hour prepractice session on Day 2 (acquired condition), there were no significant behavioural changes in prepractice session and between the last session of Day 1 and the first session of Day 2, suggesting that motor skills are still retained two weeks after initial training.

On the other hand, the mean accelerations of sessions 1–4 did not differ between the two conditions, showing that the altered motor performance did not influence dopamine release. Therefore, these behavioural results indicate that initial skill-training is associated with the intrinsic process of the acquisition of new motor skills, whereas the acquired condition is associated with the mere motor execution of the learned skill.

Based on the time-activity curves of ^11^C-raclopride (life time, 20 minutes), BP reduction reflects endogenous dopaminergic transmission related to the performance of different tasks during the PET scan, particularly just before and until several hours after the injection of ^11^C-raclopride [22], [23]. Therefore, a reduction of ^11^C-raclopride BP in the right putamen during initial skill-training compared with the acquired condition mainly reflects differences in dopaminergic transmission in session 1–4 of Day 1 versus Day 2.

ROI analysis showed that ^11^C-raclopride BP was significantly reduced in the right putamen on Day 1 compared with Day 2. Dopaminergic signals in both the striatum and motor cortex play essential roles in motor skill acquisition and the induction of synaptic plasticity [30]. In animal studies, dopaminergic signalling in the primary motor cortex is necessary for motor skill acquisition, but not for the execution of a learned task [13]. The striatal regional administration of a D1 receptor antagonist was previously shown to impair the acquisition and consolidation of motor skill memory [12], [31].

In the human, dopamine was also found to be released in the striatum during new motor sequence learning, [14], and its administration can enhance the ability to encode an elementary motor memory in the primary motor cortex [32]. Based on these findings, it is conceivable that the different patterns of dopaminergic transmission found in the right putamen reflect the encoding of new motor memory during motor skill acquisition, but not the execution of a learned skill. The study by Lappin and colleagues compared dopamine levels between the conditions of motor-sequence learning and resting, so that the task-dependent dopamine release might be contaminated by finger movements during motor-sequence learning. The present study used the acquired condition with finger movements as a control, such that possible effects of motor components could be cancelled out.

The serial condition with performance reaching nears plateau levels in Day 1 would be another important control. However, our concept in the present study was not to evaluate behavioural facilitated changes in finger movement within one day but the intrinsic processing of motor memory over a period of weeks, hence Day 2 was used as the control.

### Striatal subdivisions associated with motor skill acquisition

Voxel-wise analysis clarified sub-regions of the striatum. We observed significant BP changes in the right antero-dorsal and lateral parts of the putamen during initial skill-training compared with the acquired condition; the peak coordinate was located in the anterior putamen.

Based on anatomical and functional connectivity, the human striatum can be divided into the following functional subdivisions: the associative striatum (the caudate, anterior-dorsal and medial putamen), the sensorimotor striatum (the posterior-lateral putamen) and the limbic striatum (the ventral putamen) [33], [34]. The regions related to the skill acquisition in the present study were located in the antero-dorsal and lateral parts of the putamen, which belong to the associative and sensorimotor striatum.

Our results are consistent with previous fMRI studies that show a gradual shift of functional activation within the striatum during the initial processing of motor sequence learning; that is, a transfer of increased blood-oxygen-level-dependent (BOLD) signal from the associative to the sensorimotor striatum [35], [36], [37], [38]. This dynamic shift is observed in the early learning stage [39], [40], [41] where motor performance is improved through repeated training sessions until performance nears asymptotic levels that occur in the minutes to hours of a single training session [4], [42], [43], [44], [45]. Lehericy et al. found that the shift of activation from the anterior to the posterior putamen occurred within 10 to 50 minutes after the start of motor training [36], which is consistent with that of initial skill-training in the present study. Thus, right antero-dorsal and lateral parts of the putamen could possibly be related to the initial processing of motor memory during motor skill acquisition.

### The role of dopamine in the cortico-striatal circuit during motor skill acquisition

Regarding the cortico-striatal anatomical connection, diffusion-tensor imaging of fibre tracts has shown that the anterior-dorsal putamen connects mainly to the premotor cortex, the supplementary motor area and the prefrontal cortex. By contrast, the sensorimotor striatum connects mainly to the M1 [36], [46], [47], [48]. In accordance with the “functional gradient of inputs” to the striatum [49], [50], [51], the right antero-dorsal and lateral parts of the putamen seem to be connected to the motor-related cortex.

The functional anatomy of motor sequence learning has been extensively studied in both human and animal models, with fMRI studies revealing that the cortico-striatal circuit contributes to the early learning stage including the encoding and consolidation of a motor memory [8], [9], [10], [11], [44], [52], [53]. Moreover, previous studies have shown that dopamine selectively enhances active synapses in a task-specific manner to increase the signal-to-noise ratio [49], [54]. Therefore, the gradual shift of functional activation within the striatum during motor learning and the observed dopamine release, especially in the lateral part of the putamen, suggests that dopamine might be associated with primary motor cortico-striatal circuit activation during the formation of new motor memory.

### Dopaminergic signalling related to synaptic plasticity

The early phase of skill acquisition is probably related to synaptic plasticity in the striatum [55] [38]. Animal models have shown that the dopaminergic signal projecting from the substantia nigra is essential for inducing cortico-striatal synaptic plasticity in the striatum [38], [56], [57], [58], [59]. Furthermore, regional and training-specific changes in excitatory synaptic transmission in the striatum were recorded in brain slices from trained mice [38]. Applying TMS to patients with Parkinson's disease (PD) resulted in a dopamine-induced modification of cortical plasticity in the M1 via a motor cortico-striatal circuit [60], suggesting that such alterations to plasticity might be a physiological basis for motor skill learning in humans.

The strengthening effect of the dopaminergic signal on synaptic plasticity from the ventral tegmental area has also been reported to cause memory formation [61]. Our study findings support the idea that the cortico-striatal circuit plays a role in motor skill acquisition. This contrasts with previous work with rats that showed that the elimination of dopamine receptors and dopaminergic terminals in the prefrontal cortex and the M1 specifically impairs the induction of synaptic plasticity and motor skill acquisition [13], [62], [63]. This discrepancy could be a result of the distinct distributions of dopamine receptors in rats and the thresholds for dopamine interference with motor behaviour [13], [64], [65].

### Limitations of the study

The protocol design of this PET study has some limitations because of group comparison between two different conditions of skill acquisition. As the present study is based on acquisition of new motor skill, it is difficult to totally exclude the effect of novelty to BP changes. However, this protocol is designed to reduce novelty effect. Firstly, since novelty difference between two conditions mostly affect BP changes in session 1, subjects performed session 1 just before the PET scanning. Secondly, since it is difficult to confirm the exact time when the BP change occurred in session 1–4, they performed session 1 also inside the PET scanner (not outside) to adjust all physical and mental conditions throughout sessions. As the previous study showed, the caudate subserve the novelty effect during sequence learning task and motor responses [66]. Caudate activation was found during the learning of novel sequences of finger movements compared with those measured during performance of prelearned sequential finger movements [67]. In the present study, because we did not find ^11^C-raclopride BP changes within the caudate in ROI analysis, novelty is less likely to affect the result. Thus, we considered that the BP change in the present study is related to not novelty but encoding elementary aspects of motor behavior during skill acquisition.

In initial skill-training condition, although the mean acceleration kept increasing until session 3 against previous session, there was a slight decrease in the last session 4 against session 3. This slight decrease curve of acceleration was seen in the session 3 against session 2 also in the acquired condition. Taking it into the consideration that subjects had to perform repetitive movement relatively longer time than previous studies using the same task, even though we modified it to adjust to the PET protocol as lower frequency and longer resting times, it was likely to be caused by the variable across motor performance due to exhaustion.

Furthermore, as our hypothesis in the present study is to evaluate striatal dopamine changes in association with the encoding of new motor memory during skill acquisition compared to the acquired condition, we did not perform the direct comparison between each conditions and rest. The present study used the acquired condition with finger movements instead of rest, so that possible effects of the movement-dependent dopamine release could be cancelled out. Based on the previous study, the similar BP change was observed in the sensorimotor striatum between the conditions of motor-sequence learning and resting [14]. Therefore, findings of this study represent real intrinsic differences in dopamine release associated with the early stage of skill acquisition.

### Conclusions

We have demonstrated effects of motor skill acquisition on encoding elementary aspects of motor behavior and striatal dopamine in human. As the dopamine change in the present study was localised within the right antero-dorsal to lateral part of the putamen, our findings suggest that striatal dopamine may play a role in the dynamic cortico-striatal activation during encoding of new motor memory in skill acquisition.
